# The CaMKII‐dependent phosphorylation of GABA_B_ receptors in the nucleus accumbens was involved in cocaine‐induced behavioral sensitization in rats

**DOI:** 10.1111/cns.14107

**Published:** 2023-02-08

**Authors:** Ming F. Lu, Qiang Fu, Tian Y. Qiu, Jian H. Yang, Qing H. Peng, Zhen Z. Hu

**Affiliations:** ^1^ Department of Pathophysiology, College of Basic Medicine Nanchang University Nanchang Jiangxi China; ^2^ Jiangxi Province Key Laboratory of Tumor Pathogens and Molecular Pathology, Department of Pathology, Schools of Basic Medical Sciences and Pharmaceutical Sciences Nanchang University Nanchang Jiangxi China; ^3^ Department of Respiration, Department Two Jiangxi Provincial People's Hospital Nanchang Jiangxi China; ^4^ Department of Physiology, College of Basic Medicine Nanchang University Nanchang Jiangxi China; ^5^ Department of Anesthesiology, The First Affiliated Hospital Nanchang University Nanchang Jiangxi China

**Keywords:** behavioral sensitization, CaMKII, cocaine, GABA_B_R, nucleus accumbens, phosphorylation

## Abstract

**Background:**

Previous studies have established that the regulation of prolonged, distal neuronal inhibition by the GABA_B_ heteroreceptor (GABA_B_R) is determined by its stability, and hence residence time, on the plasma membrane.

**Aims:**

Here, we show that GABA_B_R in the nucleus accumbens (NAc) of rats affects the development of cocaine‐induced behavioral sensitization by mediating its perinucleus internalization and membrane expression.

**Materials & Methods:**

By immunofluorescent labeling, flow cytometry analysis, Co‐immunoprecipitation and open field test, we measured the role of Ca^2+^/calmodulin‐dependent protein kinase II (CaMKII) to the control of GABA_B_R membrane anchoring and cocaine induced‐behavioral sensitization.

**Results:**

Repeated cocaine treatment in rats (15 mg/kg) significantly decreases membrane levels of GABA_B1_R and GABA_B2_R in the NAc after day 3, 5 and 7. The membrane fluorescence and protein levels of GABA_B_R was also decreased in NAc GAD_67_^+^ neurons post cocaine (1 μM) treatment after 5 min. Moreover, the majority of internalized GABA_B1_Rs exhibited perinuclear localization, a decrease in GABA_B1_R‐pHluroin signals was observed in cocaine‐treated NAc neurons. By contrast, membrane expression of phosphorylated CaMKII (pCaMKII) post cocaine treatment was significantly increased after day 1, 3, 5 and 7. Baclofen blocked the cocaine induced behavioral sensitization via inhibition of cocaine enhanced‐pCaMKII‐GABA_B1_R interaction.

**Conclusion:**

These findings reveal a new mechanism by which pCaMKII‐GABA_B_R signaling can promote psychostimulant‐induced behavioral sensitization.

## Graphical abstract

This work was shown that GABA_B_ receptors (GABA_B_R) in GABAergic neurons of the nucleus accumbens (NAc) potentially regulates cocaine‐induced locomotor sensitization. Furthermore, baclofen (GABA_B_R agonist) can block cocaine‐induced locomotor sensitization via the recovery of surface expression of GABA_B_R from cocaine‐induced inhibition. Baclofen enhanced‐GABA_B_R membrane internalization and anchoring could be attributed to the suppression of cocaine‐induced enhance of interaction of *p*CaMKII–GABA_B1_R.

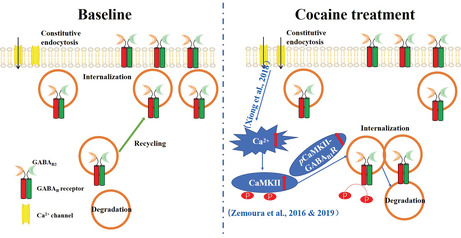

## 1 INTRODUCTION

The GABA_B_ receptor (GABA_B_R) is an obligatory heterodimer of two subunits, GABA_B1_ and GABA_B2_.^1^, ^2^, ^3^ The interaction between GABA_B1_ and GABA_B2_ facilitates its cell membrane expression through coiled–coil interactions in its cytoplasmic tail.^4^ The primary regulatory mechanism by which the GABA_B_R complex controls this inhibitory pathway is via plasma membrane availability and stability.^5^ Downregulation of the membrane expression of GABA_B_Rs in the central nervous system has been associated with many psychiatric illnesses, including epilepsy, depression, anxiety, and drug addiction.^6^ GABA_B_R is the potential target for therapeutic agents. Although a large number of antagonists and agonists and positive or negative allosteric modulators (PAMs or NAMs) have been developed for GABA_B_R,^7^ only two agonists have been approved as therapeutic drugs: baclofen used for the treatment of muscle spasticity and alcohol addiction^8^ and γ‐hydroxybutyrate (GHB) used for the treatment of narcolepsy.^9^ The mesolimbic dopamine system, in which the nucleus accumbens (NAc) is a central component, is recruited by both natural and drug reward behaviors. High‐resolution immunohistochemical imaging in the NAc identifies the enrichment of medium spiny neurons, the majority of which belong to the GABAergic neuron. Nevertheless, the molecular and cellular mechanisms of regulating GABA_B_R membrane expression in the NAc GABAergic neuron during the development of cocaine locomotor sensitization remains largely unknown.

Evidence from some studies shows that inhibition of Ca^2+^/calmodulin‐dependent protein kinase II (CaMKII), a synapse‐enriched enzyme activated when binding with Ca^2+^ and CaM in the NAc,^10^ would decrease the rewarding properties of cocaine in behavioral sensitization.^11^ The activated CaMKII autophosphorylates its own highly expressed serine/threonine site at the autoregulatory domain that further enhances the hypothesis of the interaction of CaMKII with GABA_B_R.^12^ Furthermore, activation of glutamate receptors increased CaMKII‐dependent phosphorylation in the GABA_B1_ subunit on cultured neurons and led to the endocytosis of GABA_B_R.^13^ CaMKII‐mediated phosphorylation of internalized GABA_B_R serves as a signal for K63‐linked ubiquitination of GABA_B1_ at multiple sites by the E3‐ligase Mind‐bomb 2 and sorting of the receptors to lysosomal degradation.^14^ Since phosphorylation of GABA_B1_ by the phosphorylation of CaMKII (*p*CaMKII) plays a key role in the aberrant decreased abundance of GABA_B_R under pathophysiological conditions excluding cocaine addiction, while our previous studies shown cocaine may increase locomotor activity by recruiting to dopamine D_3_ receptor (D_3_R) via the phosphorylation of CaMKII.^15^ We hypothesized that enhancing the interaction of *p*CaMKII with GABA_B1_R, and thereby upregulating phosphorylation of GABA_B1_, would induce the aberrant endocytosis and degradation of the receptors under cocaine locomotor sensitization. Baclofen (GABA_B_R agonist) should restore normal cell membrane expression of the receptors by enhancing endocytosis and recycling of receptors^16^ and block cocaine‐induced behavioral sensitization.^17^

In this study, cocaine treatment inhibited the membrane expression of GABA_B_R in NAc GAD_67_^+^ neurons. Moreover, cocaine enhanced the endocytosis of receptors and inhibited the membrane anchoring of receptors. Based on baclofen‐triggered anchoring at the membrane region of receptors, our data showed that baclofen blocked cocaine‐induced behavioral sensitization by inhibition of cocaine‐induced interaction of *p*CaMKII–GABA_B_R in NAc neurons. This study thus reveals a central role in the interaction of CaMKII–GABA_B_R during cocaine‐induced behavioral sensitization.

## 2 MATERIALS AND METHODS

### 2.1 Animals

Male Sprague–Dawley (SD) rats weighing around 250 g were used in these experiments (Committee on the Ethics of Animal Experiments of the University of Nanchang, Permit Number: 2010‐0002, RRID: RGD_728193); rats that were older or younger than 2 months (±2 weeks) were excluded. The rats were housed individually in separate cages with food and water available ad libitum. A 12‐h light/dark cycle (lights on at 7 a.m.) under constant temperature (22–27°C) was provided. The Committee on the Ethics of Animal Experiments of the University of Nanchang approved the protocol (Permit Number: 2010‐0002). All experiments were performed according to the National Institutes of Health Guide for the Care and Use of Laboratory Animals. *GAD*_*67*_‐GFP knock‐in *mice (GAD*_*67*_^*+*^ *mice)* were gifts from Dr Shujia Zhu (Institute of Neuroscience, Chinese Academy of Sciences, Shanghai 200031, China).

### 2.2 Reagents and antibodies

Antibodies were from the following companies: R&D Systems (GABA_B2_R‐AF1188 for WB and IS); Millipore (GABA_B1_R‐MABN492 for WB and IS); Kangcheng Biotechnology (GAPDH‐kc‐5G4 for WB); Sigma (pan‐Cadherin‐C1821 for WB); and Santacruz (CaMKII‐sc5306, *p*CaMKII‐sc32289, pSer‐sc81514, and GABA_B1_R‐sc398901 for co‐IP and WB). DAPI (4′,6‐diamidino‐2‐phenylindole) was from Beyotime. HRP‐conjugated IgG Fraction Monoclonal Mouse Anti‐Rabbit IgG, Light Chain Specific was from Proteintech‐SA00001‐7L. The secondary antibodies used in IS were from Invitrogen. Horseradish peroxidase‐conjugated secondary antibodies were from Millipore. GABA_B1_R‐pHluorin were constructed in this work. MYC‐GABA_B1_R were gifts from Dr Shujia Zhu (Institute of Neuroscience, Chinese Academy of Sciences, Shanghai 200031, China).

### 2.3 Measurement of locomotor activity

The experiments performed in this study followed pseudo‐randomized and repetitive measurement rules. A computer random number generator was used to launch a simple pseudo‐randomization to equally allocate the subjects to different groups. The in vivo experiments involved six rats per group and were repeated three times. (1) A total of 36 male SD rats were randomly divided into two groups (saline and cocaine, 15 mg/kg, i.p.) of six rats each in three repeated experiments. No animals died during the experiments. Open field tests were performed to measure locomotor activity for 7 consecutive days post‐treatment. (2) A total of 72 rats were randomly divided into four groups (saline + saline; saline + cocaine; baclofen + saline; and baclofen + cocaine) of six rats each within three repeated experiments. Rats received intraperitoneal (i.p.) injections of baclofen (2 mg/kg) or saline 25 min before cocaine (15 mg/kg, i.p.) or saline to measure locomotor activity following treatments. No animals died during the experiments. Open field tests were performed to measure locomotor activity for 5 consecutive days post‐treatment.

Rats were placed in an activity cage (100 × 100 × 40 cm) for 30 min for habituation 1 day before experiments. Rat locomotor activity was measured in activity cages for 10 min (Panlab Harvard Apparatus) immediately after intraperitoneal injection of cocaine (15 mg/kg, i.p.). Cages were equipped with video cameras fixed above each cage and connected to a computer equipped with SMART software (version 3.0.01; Panlab S.L.U). Rats perambulated in the activity cage for 10 min under illuminated conditions. When rat activity interrupted the infrared beams, the movement was tracked, and the connected computer collected the total traveling distance.

### 2.4 Total and membrane protein extraction

For the extraction of total protein, the tissue or cell lysates from the brain region of nucleus accumbens were prepared with RIPA buffer (CW2333; Kangwei Century Biosciences) containing phosphatase inhibitor and protease inhibitor, and the samples were centrifuged with 18495 *g* at 4°C for 10 min.

The NAc brain of rats or mice were homogenized in cold lysis buffer containing 50 mM Tris–HCl, pH 7.5, 150 mM NaCl, 1% Nonidet P‐40, 0.5% sodium deoxycholate, and protease inhibitors Cocktail set III (539134; Merck/Millipore). Membrane proteins of NAc brain of rats, mice, or cultured primary motoneurons were prepared using a plasma membrane protein extraction kit (k268‐50; Biovision) and subjected to immunoblotting experiments using indicated antibodies (see Section 2.2).

### 2.5 Western blot

After protein separation on 10% SDS‐PAGE, the protein was transferred to a polyvinylidene fluoride (PVDF) membrane. The membrane was then sealed in Tris buffer salt water containing 5% skimmed milk powder at room temperature for 2 h. The membrane was then incubated with the desired concentration of primary antibody at 4°C overnight. Primary antibodies include GABA_B1_R (1:1000), GABA_B2_R (1:1000), Pan‐cadherin (1:1000), CaMKII (1:1000), *p*CaMKII (1:1000), and *p*Ser (1:1000). The membrane was incubated at room temperature for 2 h using appropriate secondary antibodies coupled with HRP. The chemiluminescence system is used to display the chemiluminescence signals of the bands on the digital image and quantify the intensity of the bands by ImageJ software.

### 2.6 NAc cell culture, immunofluorescence, and flow cytometry

Adult pregnant female SD rats were purchased from the Central Animal Facility at Nanchang University and sacrificed by means of asphyxia in a carbon dioxide (CO_2_) chamber, according to the National Academy of Sciences' guidelines for the care and use of laboratory animals. NAc cells were removed from the brains of embryonic day 18 rats or *GAD*_*67*_^*+*^ *mice* and rinsed in ice‐cold HBSS, as described previously.^18^ Cells were incubated in 2 mL HBSS and dissociated with 2 mL trypsin–EDTA (Sigma) for approximately 12 min at 37°C. Inoculation medium (2 mL), including neurobasal growth media, 5% fetal bovine serum (FBS), 1% glutamic acid, and 1% B27, was used to terminate the dissociation following 5‐min centrifugation at 1000 r/min. NAc cells were plated at a density of 10^5^–10^6^ in an inoculation growth medium. This medium was replaced with growth medium containing neurobasal growth media, 1% glutamic acid, and 1% B27 every 3 days, and cells were used after 14 days of growth.

The 48 or 36 NAc cultures from embryonic day 18 rats and *GAD*_*67*_^*+*^ *mice* were measured after treatment with cocaine (1 μM, 5 or 10 min post‐treatments), baclofen (100 μM, 30 min post‐treatments), and saline for four cultures per group across three replicates. Membrane proteins of GABA_B1_R (1:100) and GABA_B2_R (1:100) were analyzed in the NAc cells followed by immunofluorescent labeling and flow cytometry. Briefly, for membrane staining, live neurons were incubated for 15 min at 37°C with antibodies against GABA_B1_R and GABA_B2_R. After treatments of baclofen or cocaine at 37°C, the cell slices were fixed in 4% paraformaldehyde in PBS, pH 7.4, for 10 min without permeabilization, and washed twice in PBS for 5 min. Subsequently, cell slices were incubated with 3% H_2_O_2_ at room temperature for 10 min to eliminate the bioactivity of intrinsic peroxidase and washed twice in PBS for 5 min. An appropriate amount of fluorescence‐conjugated secondary antibody (Alexa 568 and Alexa 647, 1:400; Invitrogen) was added for 120 min at room temperature, which was followed by 5 min of PBS washing. For flow cytometry analysis, cell disassociation solution was prepared with trypsin–EDTA (Sigma) and was counted. After fixing in 0.5% PFA in PBS, the cells were incubated overnight at 4°C with antibodies against GABA_B1_R and GABA_B2_R and for 120 min at room temperature. The cells were analyzed on an LSR II flow cytometer (BD Biosciences) using FACSDiva software.

### 2.7 Co‐immunoprecipitation

Based on the methods used in our lab,^15^ the interaction between *p*CaMKII and GABA_B1_R was measured using co‐immunoprecipitation. The lysate of 2 mg NAc total protein was added with 4 μg specific antibody (*p*CaMKII or GABA_B1_R) and rotated overnight at 4°C. At the same time, the same amount of protein lysate was absorbed, and the same amount of lgG of the same genus was added as the negative control group. The lysates of total protein were incubated with 80 μL Protein Ginkgo beads at 4°C for 4 h. After PBS washing five times, the precipitated complex was collected by 8000 *g* centrifugation and 10 min to obtain *p*CaMKII or GABA_B1_R protein precipitate. Add 100 μL 1× Loading Buffer, re‐suspend, and precipitate the complex, and heat it in a boiling water bath for 10 min. Next, Western blotting experiments were carried out to detect the interaction between *p*CaMKII and GABA_B1_R or between GABA_B1_R and *p*CaMKII/*p*Ser. lgG negative control group and input positive control group were set up. After the immunoblotting experiment, the gray values of each group were analyzed with Image J software, and the proportion of each group was calculated with the gray values of the target protein bands / the specific primary antibody bands. The control group was used as the control group for standardization, and then the Graphpad prism 6 software was used for statistical analysis.

### 2.8 Immunoendocytosis and image processing

Immunofluorescence, immunoendocytosis, and confocal microscopy were performed as described previously.^19^, ^20^

NAc cells were transfected using calcium phosphate, as reported elsewhere.^21^ For immunoendocytosis staining, after the transfection with MYC‐GABA_B1_R, live cells were incubated for 15 min at 37°C in media containing MYC antibodies. After washing, cells were incubated in a hypotonic medium (Dulbecco's modified Eagle's medium diluted 1:1 with water) for 5 min at 37°C, followed by isotonic KCl‐free medium for 30 min at 37°C. Live cells were left untreated or stimulated with baclofen (30 min) or cocaine (5 min). Cells were then fixed and processed for immunofluorescence. Goat serum (10% diluted in PBS) was used to block non‐specific binding for 60 min at room temperature. The surface expression of GABA_B1_R was detected with MYC and secondary antibodies (Alexa 568, 1:400; Invitrogen) prior to permeabilization. The internalized receptor pool was detected using secondary (Alexa 568, 1:400; Invitrogen) after permeabilization. For disappearing the fluorescent spots, the somas of pHluorin‐expressing neurons were stimulated with pulsed blue light (∼470 nm, 2 Hz, 5 ms per pulse, and 10 pulses per trial with 60 s intervals), followed immediately by imaging.

To quantify fluorescence intensities, confocal images were acquired using a 20× objective with identical pinhole, gain, and laser settings. A total of 24 images from three different animals from NAc cell culture and the same NAc cell culture were acquired at the same focal level. Images were acquired on a NIKON A1R or TiE laser scanning confocal microscope with 1 μm interval in each stack, reconstructed to three dimensions (3D) containing several Z‐stacks (40–50 stacks), and projected to 2D with maximum intensity using Fiji software. The membrane fluorescence image of receptors was acquired in the monolayer cell membrane with maximum intensity in each stack. For live imaging analysis, fluorescence dynamics of GABA_B1_R‐pHluorin (excitation 488 nm; EM: 500–550 nm) were obtained by recording every picture per second under a NIKON FN1 laser scanning confocal microscope (NIR Apo 40x DIC Water N.A. 0.8). Post‐acquisition images were processed with Fiji, Adobe Photoshop CC 2017, and Illustrator CS5 software. Data were shown as means ± SEM from at least three experiments (*p* ≤ 0.05 was considered a significant difference).

### 2.9 Quantification and statistical analysis

Student's *t*‐tests (unpaired), Pearson correlation analysis, and one‐way ANOVA tests were used to determine statistical differences for behavioral data using GraphPad Prism 6 (Graphpad Software) or SPSS 20.0. Dunnett's post hoc analysis or Bonferroni's post hoc test was applied when an ANOVA showed a significant main effect. Statistical significance was *p* < 0.05. All data are presented as means ± SEM.

## 3 RESULTS

### 3.1 The membrane expression of GABA_B_R was decreased in NAc GABAergic neurons post‐cocaine treatment

**FIGURE 1:**
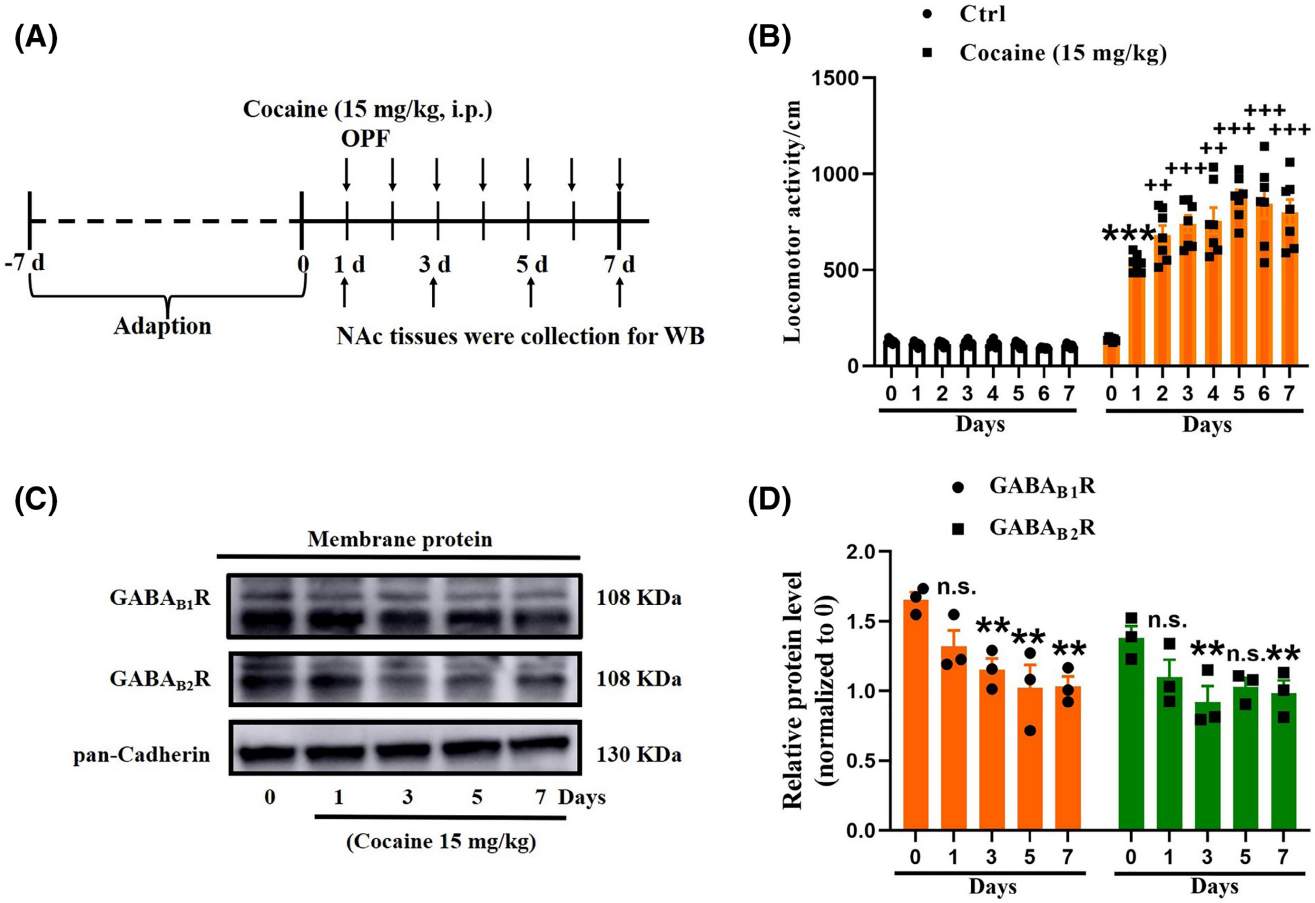
Cocaine treatments induce locomotor activity in rats by decreasing membrane expression of GABA_B_R in NAc region. (A) Experimental timeline of the cocaine‐induced behavioral sensitization in rats. (B) Quantification of the total distance traveled for 10 min by saline or cocaine (15 mg/kg) in the open field test on 7 consecutive days. Effects of cocaine on behavioral sensitization were observed on day 2 and continued to day 7. The data are the means ± SEM of at least six rats. ***p < 0.001 compared with the saline group [one‐way analysis of variance (ANOVA), followed by Dunnett's post hoc test]. ^++^ *p* < 0.01 and ^+++^ *p* < 0.001 compared with cocaine‐treated group on day 1 (one‐way ANOVA, followed by Dunnett's post hoc test). (C, D) Representative membrane protein Western blotting (WB) of GABA_B1_R and GABA_B2_R obtained at 0, 1, 3, 5, and 7 days post‐cocaine treatment. The quantification of the relative optical density (OD) of GABA_B_R versus pan‐cadherin with significant differences of comparisons using one‐way ANOVA followed by Dunnett's post hoc test. The data are the means ± SEM of three rats. **p < 0.01 compared with the saline group.

To investigate the role of GABA_B_R in cocaine addiction, Sprague–Dawley (SD) rat locomotor activity was measured using open field tests immediately after treatment with saline or cocaine for 7 consecutive days. The repeated administration of cocaine (15 mg/kg) resulted in significantly increased locomotor activity on Day 1, and continued to increase relative to this level through Day 2, indicating the onset of behavioral sensitization, and peaked on Day 5 (Figure 1B). These results were consistent with previously published findings.^15^, ^22^, ^23^ Therefore, we next examined the patterns of GABA_B_R expression at the cell membrane in rat NAc tissue during the early stages of cocaine addiction. Since functional GABA_B_Rs are obligatory heterodimers composed of GABA_B1_ and a second heptahelical membrane protein, GABA_B2_, which shares ~35% sequence identity with GABA_B1_,^24^, ^25^ we also examined the expression of these individual subunits. The results showed that membrane levels of GABA_B1_R and GABA_B2_R significantly declined on the 3rd, 5th, and 7th days of cocaine treatment compared to those of the control group (Figure 1C,D).

**FIGURE 2:**
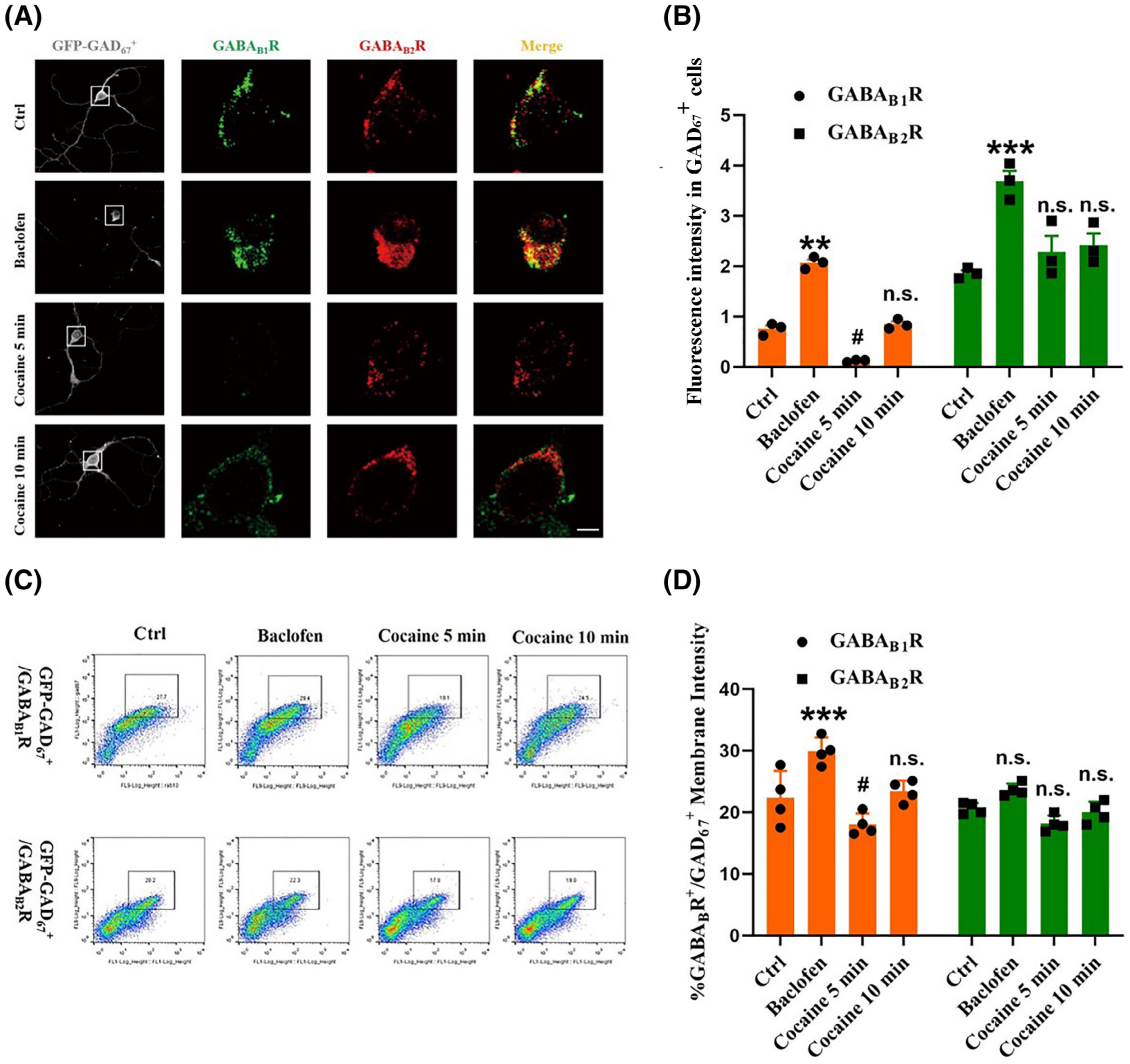
Cocaine treatments inhibit membrane expression of GABA_B_R in GAD_67_^+^ NAc neurons. (A, B) GAD_67_^+^ NAc cells were stained with antibodies against GABA_B1_R (green), GABA_B2_R (red), and GFP (gray). Scale bar: 10 μm. Cells were treated with saline, baclofen (100 μM), or cocaine (1 μM). Data are means ± SEM of three GAD_67_^+^ mice. Dunnett's post hoc test was used to determine significance. ***p < 0.001; **p < 0.01; and ^#^ *p* < 0.05 compared with the control group. Ctrl, control; n.s., non‐significant. (C, D) NAc cells of GAD_67_^+^ mice were subjected to flow cytometry (FC) with antibodies against GABA_B1_R, GABA_B2_R, and GAD_67_^+^. Cells were treated with saline, baclofen (100 μM), or cocaine (1 μM). Data are means ± SEM of four GAD_67_^+^ mice. Dunnett's post hoc test was used to determine significance. ***p < 0.001 and ^#^ *p* < 0.05 compared with the control group. Ctrl, control; n.s., non‐significant.

It is well established that the principal cell types in the NAc are GABAergic neurons. We next investigated the cocaine‐induced GABA_B_R membrane expression in NAc GAD_67_^+^ neurons using baclofen treatment as a positive control, with confocal microscopy and flow cytometers. We observed that immunofluorescence of GABA_B_R membrane levels in NAc GAD_67_^+^ neurons was increased under baclofen treatments, as expected, but GBAB_B1_R levels were decreased within 5 min of cocaine treatment (Figure 2A,B). These results were confirmed by flow cytometry analysis of membrane‐associated signals (Figure 2C,D), as well as decreased protein levels of GABA_B1_R and GABA_B2_R within 5 min of cocaine treatments.

### 3.2 The perinucleus internalization of GABA_B_R was triggered in NAc neurons post‐cocaine treatments

**FIGURE 3:**
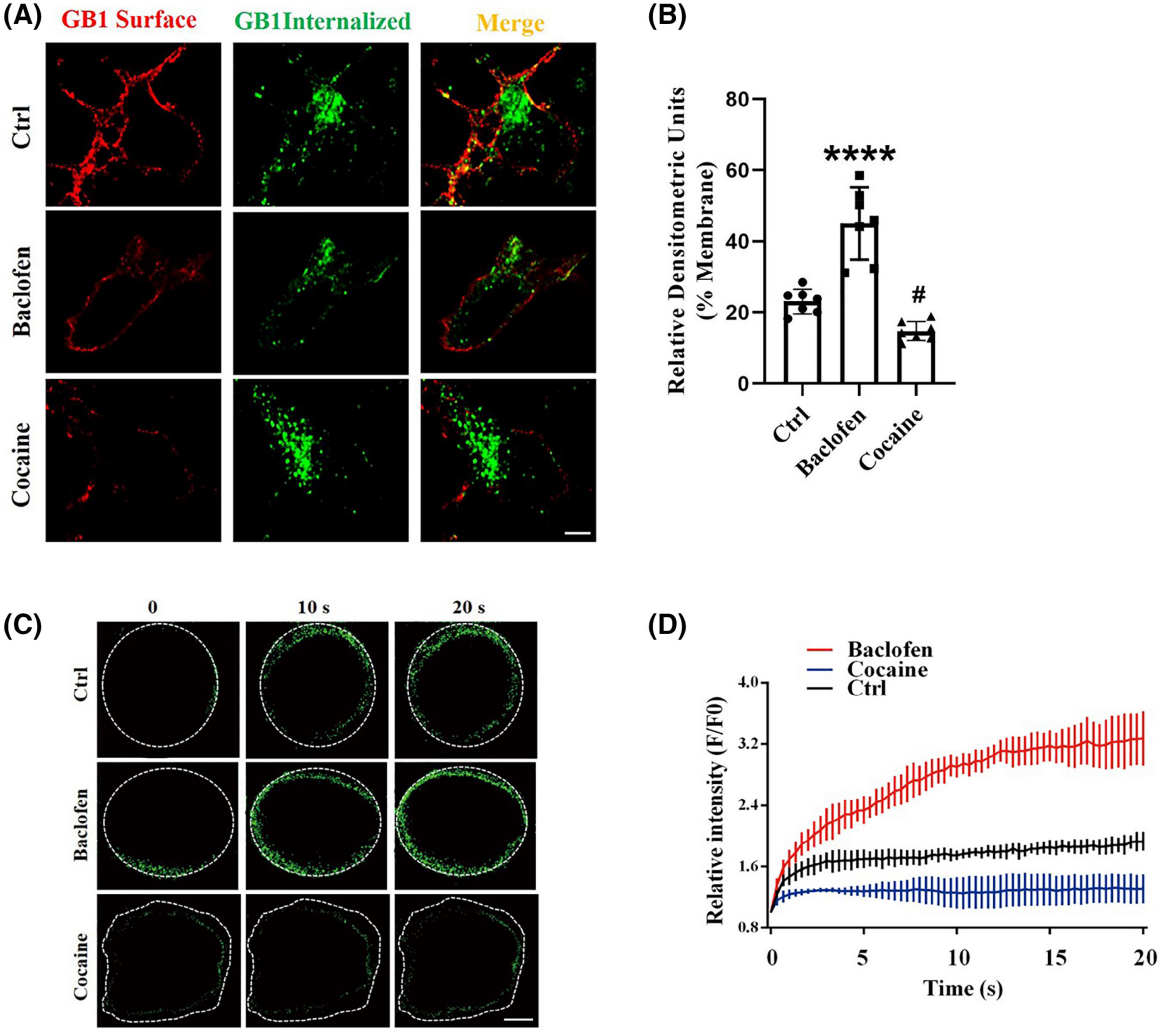
The activity‐associated internalization of GABA_B_R was triggered in NAc neurons post‐cocaine treatment. (A, B) NAc neurons were transfected with MYC‐ GABA_B1_R and processed for immunoendocytosis with MYC antibodies. Cells were left untreated (Upper), exposed to 100 μm baclofen for 30 min (Middle), or exposed to 1 μM cocaine for 5 min (Lower). Membrane receptors were detected with MYC‐ and Texas Red‐conjugated secondary antibodies (red channel). Internalized receptors were detected using FITC‐conjugated secondary antibodies (green channel). (Right) Merged images are shown. Scale bar: 10 μm. Data are means ± SEM of seven cells from three mice. Dunnett's post hoc test was used to determine significance. ****p < 0.0001; ^#^ *p* < 0.05 compared with the control group. Ctrl, control; ns, non‐significant. (C, D) NAc neurons transfected with GABA_B1_R‐pHluorin were stimulated with ~470 nm laser, followed by time lapse imaging. Note the decrease in the fluorescence intensity of GABA_B1_R‐pHluorin on the membrane of cells caused by photobleaching. White dash line: cells membrane. Scale bar: 10 μm. Normalized fluorescence intensity of GABA_B1_R‐pHluorin. Data presented are the mean value of 10 cells with SEM of three mice.

Based on the results of GBAB_B1_R significant decline in NAc GAD_67_^+^ neurons under cocaine treatments, we next used immunostaining in NAc neurons through labeling of the GBAB_B1_R subunit to directly visualize the cocaine‐induced GABA_B_R endocytosis and degradation process (Figure 3A,B). Confocal microscopy showed that GABA_B_Rs appeared to accumulate throughout the cell, including the neuronal membrane and perinuclear regions. Specifically, GABA_B1_R internalization was also clearly observed at the neuronal membrane after baclofen stimulation, whereas after cocaine stimulation, the majority of internalized GABA_B_Rs exhibited perinuclear localization, which was consistent with our results showing decreased GABA_B1_R at the cell membrane under cocaine treatment.

To further test this hypothesis, NAc neurons were transfected with an expression vector encoding GABA_B1_R tagged with pHluorin, which exhibits relatively low fluorescence in the acidic vesicle lumen but produces higher fluorescence signals when fused with the plasma membrane and is exposed to higher pH on the external cell membrane. We then measured the GABA_B1_R‐pHluorin signal to characterize the effects of cocaine on GABA_B_R activity in membrane anchoring. We found that baclofen‐activated neurons exhibited a marked increase in GABA_B1_R‐pHluorin signals, although a decrease in GABA_B1_R‐pHluorin signals was observed in the cocaine‐treated neurons (Figure 3C,D). Taken together, these findings provided direct evidence that cocaine inhibits GABA_B_R membrane expression via triggering receptor internalization at the perinuclear region.

### 3.3 GABA_B_R was involved in regulating rat cocaine‐induced behavioral sensitization

**FIGURE 4:**
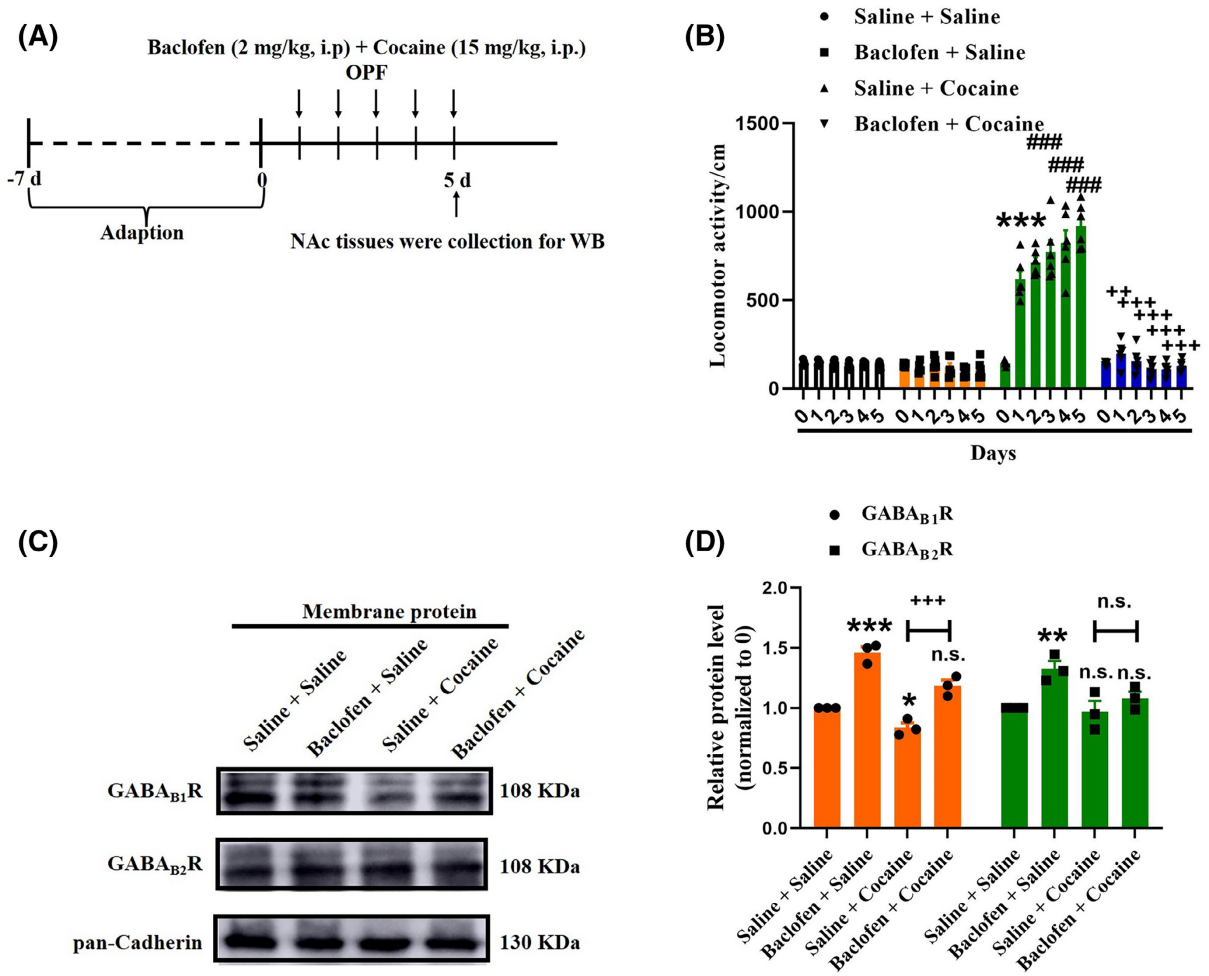
GABA_B_R was involved in regulating rat cocaine‐induced behavioral sensitization. (A) Experimental timeline of the cocaine‐induced behavioral sensitization in rats of pretreatment with baclofen. (B) Quantification of the total distance traveled for 10 min by treatments with saline, baclofen, or cocaine in the open field test on 5 consecutive days. The data are the means ± SEM of six rats. ***p < 0.001 compared with the saline group [two‐way analysis of variance (ANOVA), followed by Dunnett's post hoc test]. ^###^ *p* < 0.001 compared with cocaine‐treated group on day 1 (two‐way ANOVA, followed by Dunnett's post hoc test). ^++^ *p* < 0.01 and ^+++^ *p* < 0.001 compared with the cocaine group (two‐way ANOVA, followed by Dunnett's post hoc test). (C, D) Representative membrane protein WB of GABA_B1_R and GABA_B2_R obtained from three mice after post‐treatments with saline, baclofen, or cocaine. The quantification of the relative OD of GABA_B_R versus pan‐cadherin with significant differences of comparisons using two‐way ANOVA followed by Dunnett's post hoc test. *p < 0.05, **p < 0.01, and ***p < 0.001 compared with the saline group. ^+++^ *p* < 0.001 compared with the cocaine group (two‐way ANOVA, followed by a post hoc *t*‐test). n.s., non‐significant.

Several studies have indicated that GABA_B_R participates in cocaine treatment. Thus, to identify whether the GABA_B_R responsible is for cocaine‐induced behavioral sensitization, baclofen (GABA_B_R agonist) was injected intraperitoneally (2 mg/kg, i.p.) 5 days prior to treatment with cocaine or saline (Figure 4A). Open field tests of data collected for 5 consecutive days post‐baclofen‐injection treated with saline exhibited similar locomotor activity compared to that of saline injection control rats (Figure 4B), which suggested that baclofen alone had no obvious effect on locomotor activity in saline‐treated physiological conditions in vivo. Furthermore, in the cocaine treatment groups, measurement of the total traveling distance revealed a significant increase on day 2 compared with on day 1 as well as development of behavioral sensitization on day 2 (Figure 4B). Although the post‐baclofen‐injection rats exhibited a significant decrease in total traveling distance compared with that of the cocaine‐treated rats, they showed less sensitivity to cocaine, suggesting that GABA_B_R agonist could block cocaine‐induced behavioral sensitization.

We also collected WB analysis of samples at 5 days post‐injection and analyzed membrane expression of GABA_B_R in NAc regions and found that membrane protein levels of GABA_B1_R and GABA_B2_R were significantly higher in the NAc neurons treated with baclofen injection than those in saline injection neurons (Figure 4C,D). Moreover, after cocaine treatments, membrane protein levels of GABA_B1_R were significantly higher in the neurons treated with baclofen‐injected compared to that in neurons treated with saline‐injected (Figure 4C,D). Taken together, these findings provided further evidence that GABA_B_R in the NAc region was involved in the process of cocaine‐induced behavioral sensitization.

### 3.4 CaMKII–GABA_B_R was involved in regulating rat cocaine‐induced behavioral sensitization post‐treatments with baclofen injection

**FIGURE 5:**
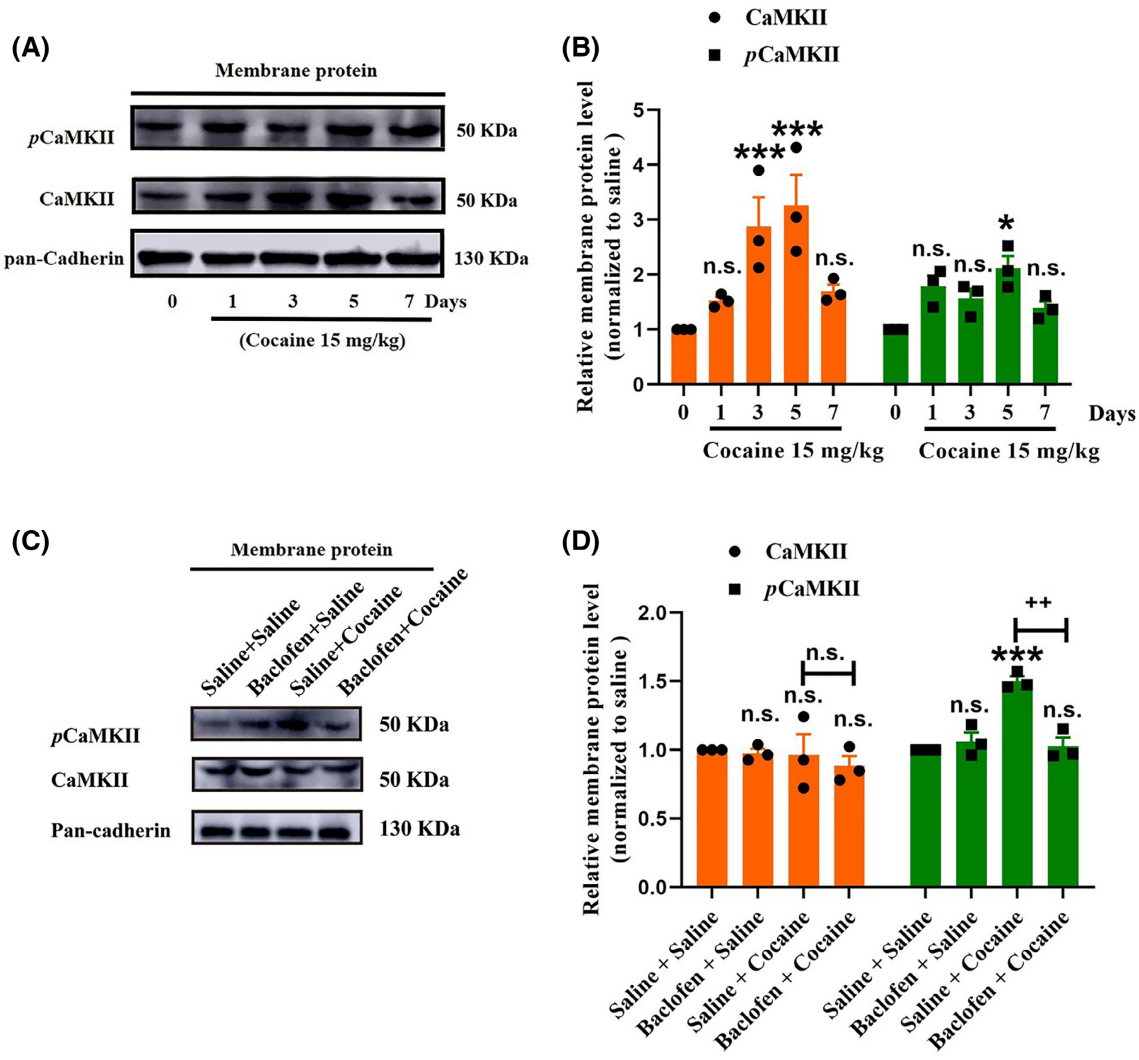
CaMKII was involved in GABA_B_R‐regulating rat cocaine‐induced behavioral sensitization. (A, B) Representative membrane protein WB of CaMKII and *p*CaMKII obtained on the days 1, 3, 5, and 7 post‐cocaine treatment. The quantification of the relative OD of CaMKII and *p*CaMKII versus pan‐cadherin with significant differences of comparisons using one‐way ANOVA followed by Dunnett's post hoc test. Data are means ± SEM of three mice. *p < 0.05 and ***p < 0.001 compared with the saline group. n.s., non‐significant. (C, D) Representative membrane protein WB of GABA_B1_R and GABA_B2_R obtained post‐treatment of saline, baclofen, or cocaine. The quantification of the relative OD of GABA_B_R versus pan‐cadherin with significant differences of comparisons using two‐way ANOVA followed by Dunnett's post hoc test. ***p < 0.001 compared with the saline group. ^++^ *p* < 0.01 compared with the cocaine group (two‐way analysis of variance (ANOVA), followed by a post hoc *t*‐test). n.s., non‐significant.

Previous studies have established that the critical mechanism of surface membrane expression of GABA_B_R is intensely associated with the status of GABA_B1_R phosphorylation modulated by calcium‐/calmodulin‐dependent protein kinase II (CaMKII).^13^, ^14^, ^26^ We measured the membrane expression of CaMKII and *p*CaMKII in the NAc tissue of SD rats on days 1, 3, 5, and 7 of cocaine treatment combined with baclofen injection. Both CaMKII and *p*CaMKII expression showed significant changes in their membrane expression levels induced by treatments (Figure 5A,B). Specifically, membrane expression levels of *p*CaMKII increased after cocaine treatment compared with saline treatment (Figure 5A,B), by contrast to which GABA_B_R membrane expression decreased (Figure 1C,D). In addition, although there was no statistically significant difference in the membrane expression of CaMKII in rat NAc tissue on the Day 5 of treatments from groups (Figure 5C,D), treatment with baclofen restored the normal membrane expression of *p*CaMKII on the Day 5 of cocaine treatment (Figure 5C,D). These data thus supported an association between *p*CaMKII and GABA_B_R during cocaine addiction. We hypothesized that GABA_B_R triggered a complex signal cascade that leads to cocaine locomotor sensitization, most likely through CaMKII signaling.

**FIGURE 6:**
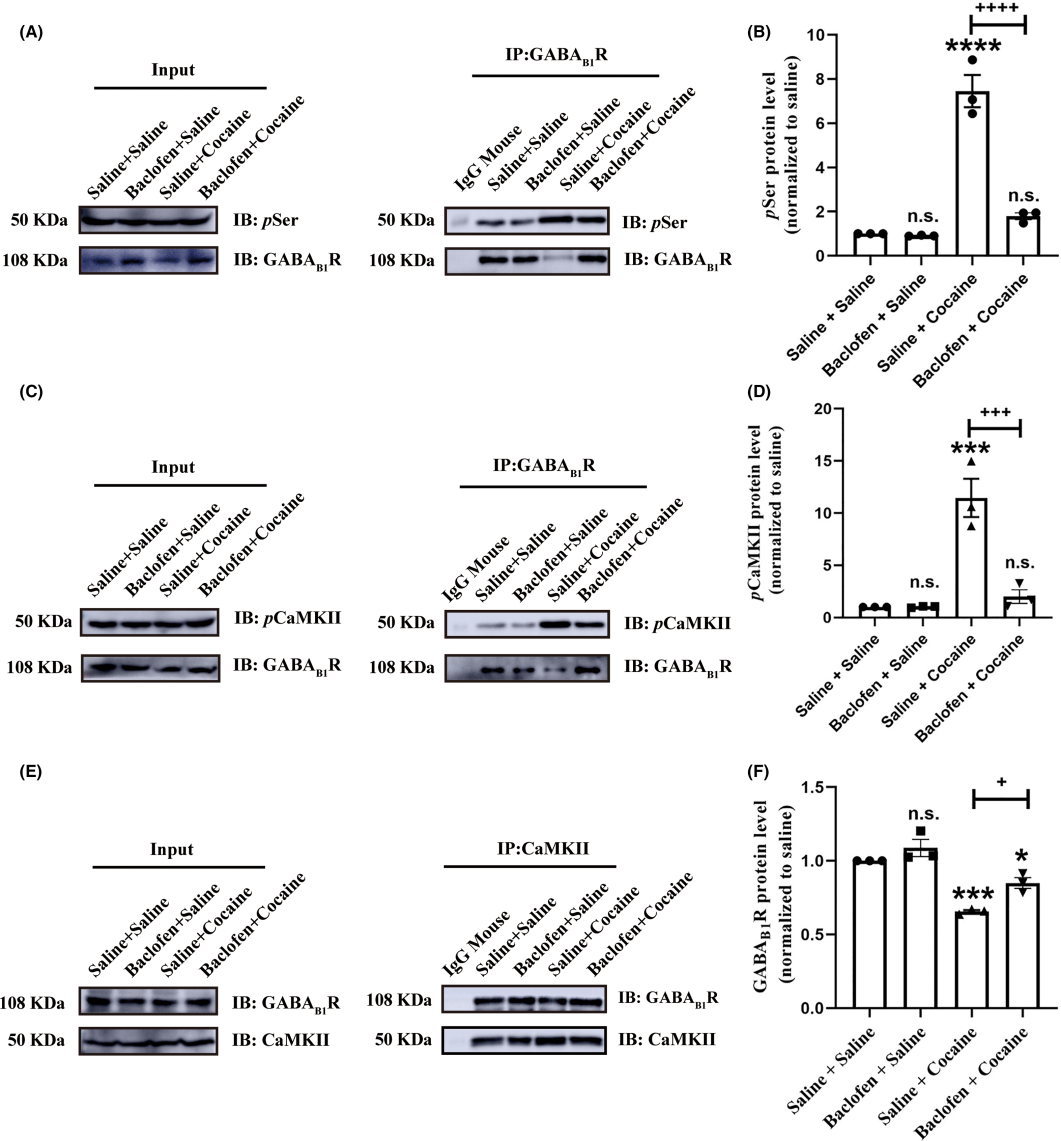
The interaction of CaMKII and GABA_B1_R was involved in baclofen‐regulating rat cocaine‐induced behavioral sensitization. (A) The same NAc brain lysate was pulled down with GABA_B1_R‐specific antibody for immunoprecipitates (IP) and Western blotting (WB), and the *p*Ser expression was measured in GABA_B1_R immunoprecipitates. (B) The quantification of the relative OD of *p*Ser versus immunoprecipitates (GABA_B1_R) with significant differences of comparisons using two‐way ANOVA followed by Dunnett's post hoc test. Data are means ± SEM of three mice. ****p < 0.0001 compared with the saline group, ^++++^ *p* < 0.0001 compared with the cocaine group; n.s., non‐significant. (C) The same NAc brain lysate was pulled down with GABA_B1_R‐specific antibodies for IP and WB, and the *p*CaMKII expression was measured in GABA_B1_R immunoprecipitates. (D) The quantification of the relative OD of *p*CaMKII versus immunoprecipitates (GABA_B1_R) with significance differences of comparisons using two‐way ANOVA followed by Dunnett's post hoc test. Data are means ± SEM of three mice. ***p < 0.001 compared with the saline group, ^+++^ *p* < 0.001 compared with the cocaine group; n.s., non‐significant. (E) The same NAc brain lysate was pulled down with *p*CaMKII‐specific antibody for IP and WB; the GABA_B1_R expression was measured in *p*CaMKII immunoprecipitates (F) The quantification of the relative OD of GABA_B1_R versus immunoprecipitates (*p*CaMKII) with significant differences in comparisons using two‐way ANOVA followed by Dunnett's post hoc test. Data are means ± SEM of three mice. *p < 0.05 and ***p < 0.001 compared with the saline group, ^+^ *p* < 0.05 compared with the cocaine group; n.s., non‐significant.

CaMKII masterminds the neuroadaptation of GABA_B_Rs via straight autophosphorylating the S867 of GABA_B1_ in the hippocampus to trigger endocytosis and degradation.^13^ In order to determine whether phosphorylation of CaMKII participates in GABA_B_R regulation of the cocaine‐induced behavioral sensitization process, we again analyzed the interaction of *p*CaMKII*–*GABA_B1_R by co‐immunoprecipitation in the NAc. In the cocaine treatment group, the phosphorylation of GABA_B1_R (Figure 6A,B) and *p*CaMKII expression (Figure 6C,D) in GABA_B1_R immunoprecipitates was increased, by contrast to which the GABA_B1_R expression was decreased in *p*CaMKII immunoprecipitates compared to saline treatment group (Figure 6E,F). These findings suggested that cocaine enhanced the interaction of *p*CaMKII–GABA_B1_R in the NAc region. However, baclofen inhibited cocaine‐increased *p*CaMKII–GABA_B1_R interaction in NAc regions (Figure 6A–F). During the process of cocaine‐induced *p*CaMKII–GABA_B1_R interaction, *p*CaMKII binds to GABA_B1_R, phosphorylates GABA_B1_R at S867 residue site, and decreased the membrane expression of GABA_B_R via triggering receptors internalization at the perinucleus region. These findings indicated that the enhanced CaMKII phosphorylation associated with GABA_B1_R phosphorylation results in less GABA_B_R membrane expression under cocaine addiction than under normal physiological conditions.

## 4 DISCUSSION

Our previous studies demonstrated cocaine locomotor sensitization could be blocked after microinjection of the cocaine‐ and amphetamine‐regulated transcript (CART) peptide into the NAc regions,^27^ which significantly rescued the cocaine‐inhibited membrane expression of GABA_B_R in NAc tissues (data not shown). GABA_B_R in the NAc regions is a major regulatory protein controlling the behavioral effects of psychostimulants.^28^, ^29^ However, the membrane expression patterns of GABA_B_R during exposure to cocaine and its potential inhibitory effects (and mechanisms underlying these effects) on cocaine addiction have remained poorly understood. The results presented in this work suggest that CaMKII participates in mediating GABA_B_R membrane expression in cocaine locomotor sensitization. This conclusion is supported by several lines of evidence: (1) differential membrane expression analyses indicated that GABA_B_R in the NAc neurons was significantly downregulated by enhancing its perinucleus internalization and inhibiting its membrane anchoring during the repeated response to cocaine; (2) GABA_B_R agonist (baclofen) upregulated membrane expression of GABA_B_R and stimulated its membrane internalization and anchoring; and (3) most importantly, baclofen blocked cocaine‐induced behavioral sensitization via inhibiting the cocaine‐enhanced interaction of *p*CaMKII–GABA_B1_R in the NAc region.

Under addiction conditions induced by psychostimulants, the “long‐loop” GABAergic feedback is mediated primarily by inhibition of GABA_B_Rs in NAc neurons.^30^, ^31^ We report here that repeated treatments of cocaine lead to the decrease in membrane expression of GABA_B_R in NAc neurons. Baclofen blocked cocaine locomotor sensitization via the increase in membrane expression of GABA_B_R. These data are consistent with the observation that cocaine has been shown to suppress GABA_B_R‐dependent function in NAc neurons in vivo,^30^, ^32^ and that methamphetamine self‐administration suppresses GABA_B_R‐dependent responses in GABA neurons of VTA.^29^, ^33^ Treatment with baclofen, a selective GABA_B_R agonist, attenuates cocaine‐induced dopamine (DA) efflux into the NAc and is accompanied by decreased psychostimulant‐induced hyperlocomotion, self‐administration, and conditioned place preference (CPP).^34^, ^35^, ^36^ Moreover, clinical and preclinical studies of addiction indicate that baclofen attenuates drug‐seeking behavior, drug craving, and relapse.^34^ Given the central roles of GABA_B_R in mediating long‐lasting neuronal inhibition, their activity is at least partially determined by their stability on the plasma membrane. Based on this body of evidence, we hypothesized that addiction‐associated behaviors may be induced by low membrane availability due to the poor stability of GABA_B_R.

The psychostimulant‐evoked reduction in membrane expression of GABA_B_R in NAc GABA neurons could stem from an impairment of regulation and balance in G protein coupling^33^ or constitutively internalization of the receptor in the membrane or perinucleus region.^37^ Our data showed quantitative immunofluorescence confocal microscopy revealed a significant reduction in membrane internalization and membrane expression of GABA_B_R in GABA neurons of cocaine treatments rats, coincident with a decrease in expression of GABA_B1_R in the *p*CaMKII immunoprecipitates. Previous studies have established that the phosphorylation of GABA_B1_R is regulated by Ca^2+^/calmodulin‐dependent protein kinase II (CaMKII), and that prolonged activation of NMDA‐triggered endocytosis of GABA_B_R occurs via activation of CaMKII and phosphorylation of the GABA_B1_ subunit.^13^ Pharmacological blockade of CaMKII phosphorylation significantly increases the cell membrane expression of GABA_B_R.^14^ Indeed, we found that repeated exposure to cocaine‐induced *p*CaMKII immunoprecipitated improved the phosphorylation of GABA_B1_R, and thereby perinucleus internalization of the receptor in the NAc.^13^ In addition, phosphorylation of GABA_B1_R by CaMKII recruits the E3 ubiquitin ligase MIB2 resulting in the K63‐linked ubiquitination of the receptor at multiple sites, which tags the receptors for lysosomal degradation.^38^ Blocking recycling results in fast degradation of the receptors, whereas inhibition of lysosomal degradation favors recycling and increases cell membrane expression. During cocaine locomotor sensitization, this reconstitutes recycling of the receptors and, together with the delivery of newly synthesized receptors, depresses cell membrane GABA_B_R expression levels. The persistence of the cocaine‐induced GABA_B_R depression suggests the balance of membrane and internalized GABA_B_R in GABA neurons might be controlled by a molecular switch in a phosphatase, perhaps akin to the autophosphorylation switch in CaMKII.

In the brain, CaMKIIα and CaMKIIβ are the predominant subunits. T286‐autophosphorylated CaMKIIα stimulates CaMKII activity by Ca^2+^ dependent, which results in glutamate receptor trafficking to the cell membrane.^13^ CaMKIIα is also a key contributor to the behavioral effects of psychostimulants.^11^, ^39^ As the levels of CaMKII activity are regulated by the intracellular concentration of Ca^2+^, Ca^2+^/CaMKII is ideally positioned to link increased neuronal activity post‐cocaine treatment with the downregulation of GABA_B_R membrane expression. Our previous data have shown that the lentivirus (LV)‐CaMKIIα rats exhibited higher locomotor activity and followed a much more chaotic movement track than the LV‐negative rats. Measurement of the total traveling distance revealed an obvious increase in locomotor activity in cocaine‐treated LV‐negative rats.^15^ We, therefore, hypothesized that the mechanism underlying cocaine‐mediated inhibition of GABA_B_R activity is associated with Ca^2+^/CaMKIIα signaling.^19^, ^40^ In contrast, it is previously reported that upregulation of CaMKIIβ, but not CaMKIIα, decreases the membrane protein of GABA_B_R by increasing the interaction GABA_B_R with CaMKII,^14^ the exact subunits of CaMKII involved in phosphorylating GABA_B1_R in procession of cocaine‐induced behavioral sensitization need further study. In addition, normal expression levels and activity of CaMKII are fundamental to neuronal physiology. CaMKII regulates a variety of intracellular processes important for neuronal homeostasis and plays a central role in controlling synaptic plasticity. Therefore, it was important that injection of baclofen did not affect CaMKII expression under physiological conditions to avoid undesired side effects. However, cocaine treatments increased autophosphorylation of CaMKII, which renders it persistently active.

We also asked what mechanism underlies the cocaine‐evoked depression in membrane expression of GABA_B_R in NAc GABA neurons alter the physiology of the NAc and contribute to addiction? The main neuronal population of NAc are the GABAergic medium spiny neurons, which form the main efferent projection from NAc, and comprise around 95% of accumbal neurons.^41^ γ‐aminobutyric acid (GABA) is the major inhibitory neurotransmitter in the central nervous system, and GABA_B_R mediates the slow and prolonged phase of inhibitory post‐synaptic potentials.^42^, ^43^ One possibility stems from the observation that GABA_B_R directly mediated mechanisms exert local inhibitory control over dopamine release in NAc. Addition possibility arises from the observation that GABA_B_R trafficking is under direct control of NMDA‐mediated systems,^17^ and disruption could provide plausible mechanism through which repeated psychostimulant treatment may affect GABA_B_R function. In this context, it will be beneficial to test the effects of GABA_B_R‐specific drugs on disruption of behaviors resembling locomotor sensitization. Although the exact mechanism of GABA_B_R dysfunction remains uncertain and warrants further study, it nevertheless provides a potential route for the therapeutic treatment of cocaine addiction, either alone or as an adjunct to current treatment.

In conclusion, our study uncovers an important feedback role for CaMKII in mediating GABA_B_R membrane expression during cocaine‐induced behavioral sensitization. Nevertheless, this study does not exclude the involvement of other factors that contribute to GABA_B_R membrane anchoring in neurons after treatment with cocaine. It remains possible that other kinases are also involved, both AMP‐activated protein kinase (AMPK)‐dependent phosphorylation of GABA_B_R and PP2A‐dependent dephosphorylation govern post‐endocytic sorting of GABA_B_R.^17^ Hence, the restoration of GABA_B_R membrane anchoring and membrane expression following treatments of cocaine combined with baclofen is a promising approach, as it interferes with a critical element of autonomous activity of CaMKII.

## AUTHOR CONTRIBUTIONS

ZZH designed the research, QF, MFL, JHY, TYQ, and QHP performed the research; QF and ZZH analyzed data. ZZH and MFL wrote the study. All authors have read ad agreed to the published version of the manuscript.

## CONFLICT OF INTEREST STATEMENT

The authors declare no conflict of interest.

## Supporting information


Figures S1–S6

## Data Availability

The data presented in this article or Supporting Information are available.
