# Supplementary material for: The CaMKII‐dependent phosphorylation of GABAB receptors in the nucleus accumbens was involved in cocaine‐induced behavioral sensitization in rats
Source: CNS Neurosci Ther. 2023 Feb 8;29(5):1345–56. doi: 10.1111/cns.14107 (PMC10068462; doi:10.1111/cns.14107)

Figure 1 B Pan-cadherin

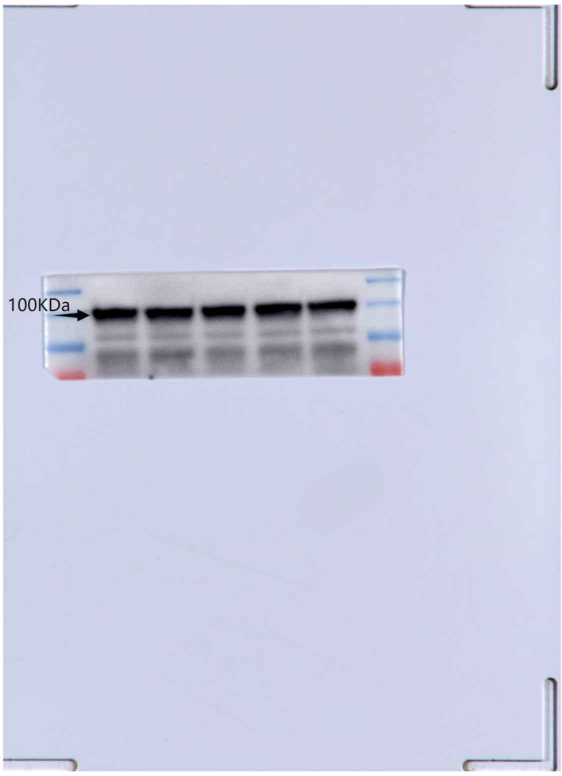

Figure 1 B GABA$_{B2}$R

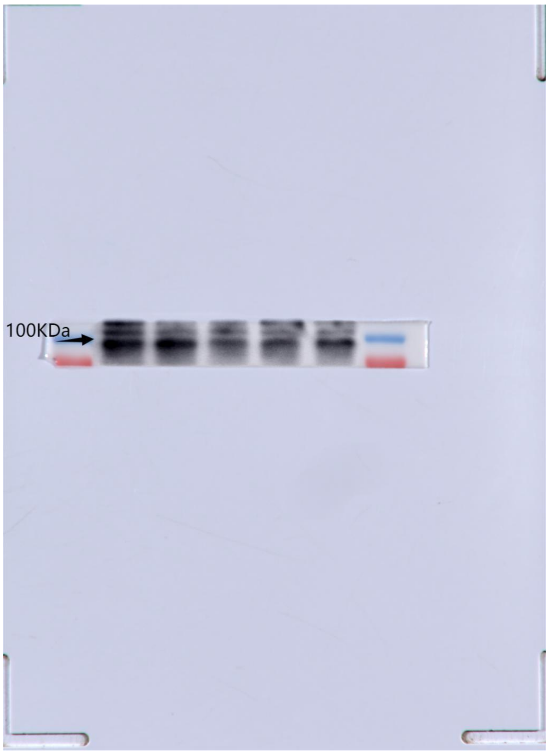

Figure 1 B GABA$_{B1}$R

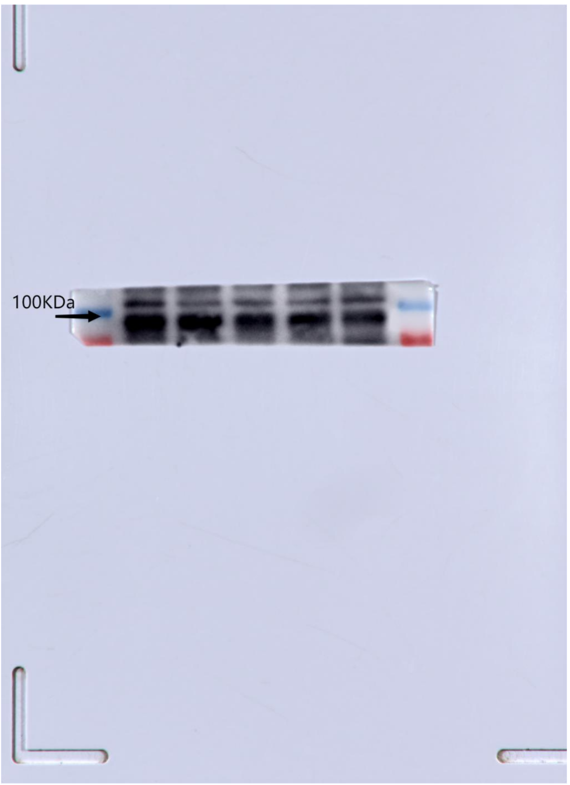

Figure 4 C

Pan-Cadherin

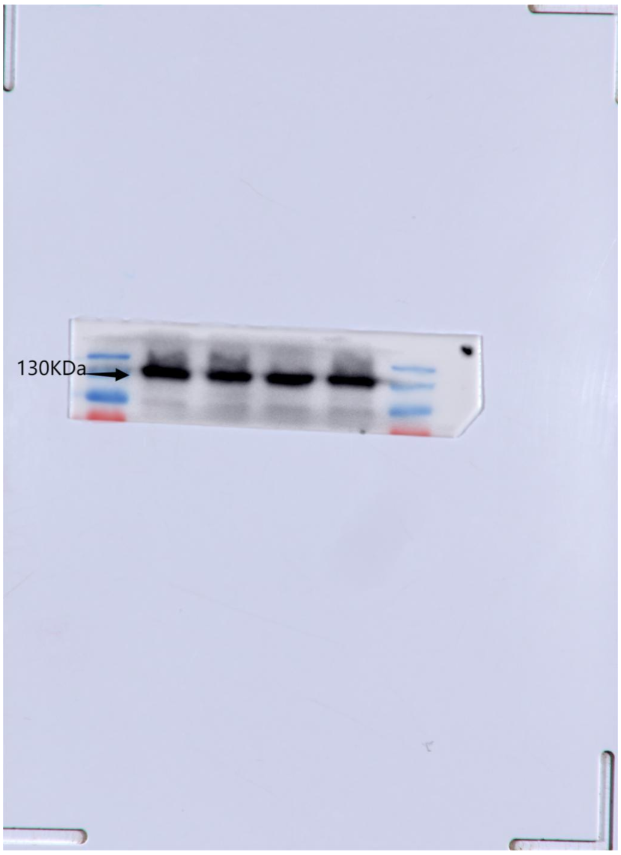

$GABA_{B1}R$

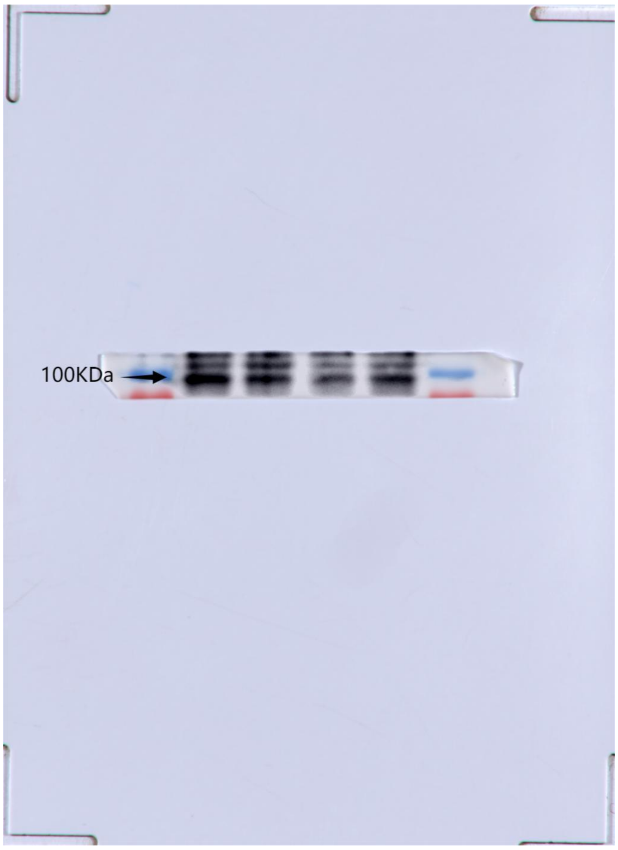

$GABA_{B2}R$

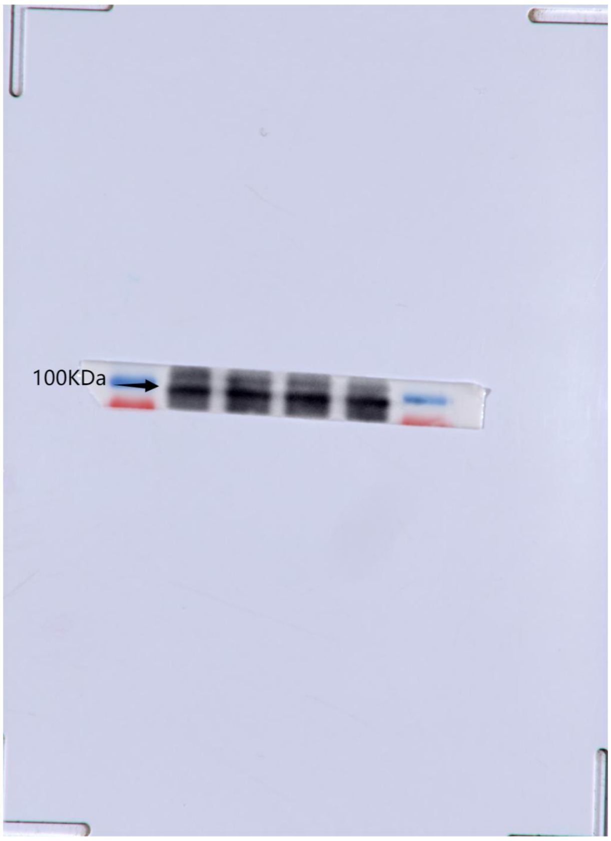

Figure 5 A

Pan-cadherin

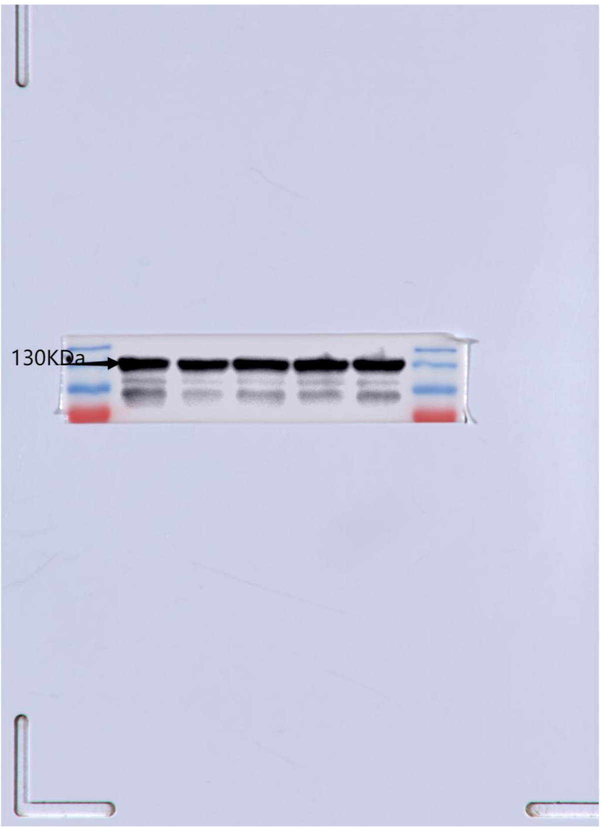

CaMKII

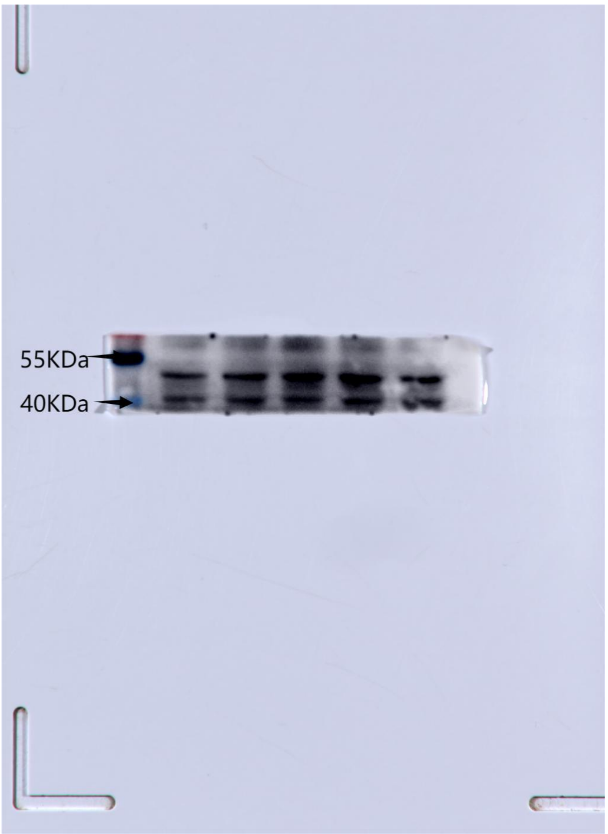

p-CaMKII

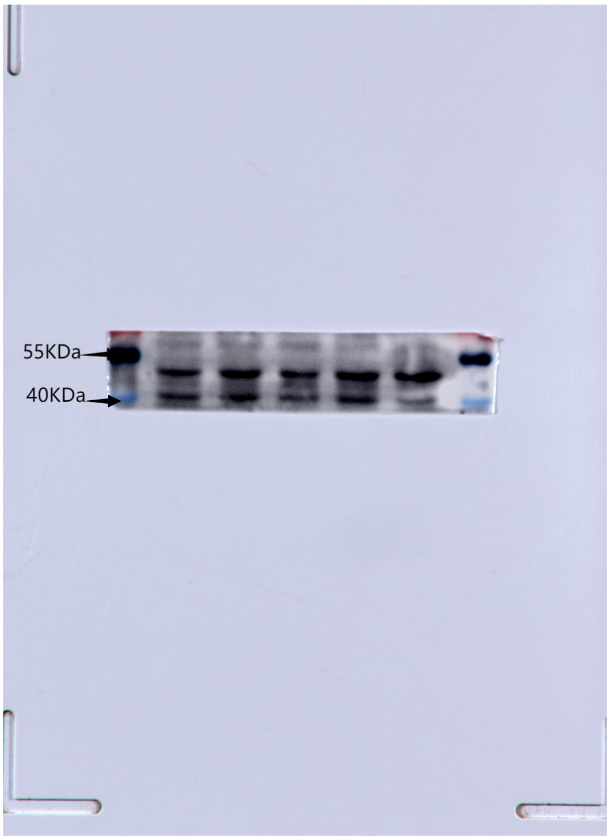

Figure 5 C

Pan-cadherin

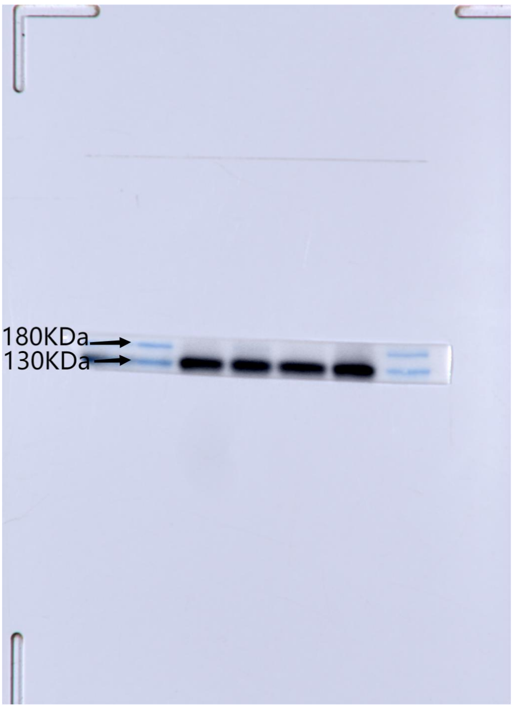

CaMKII

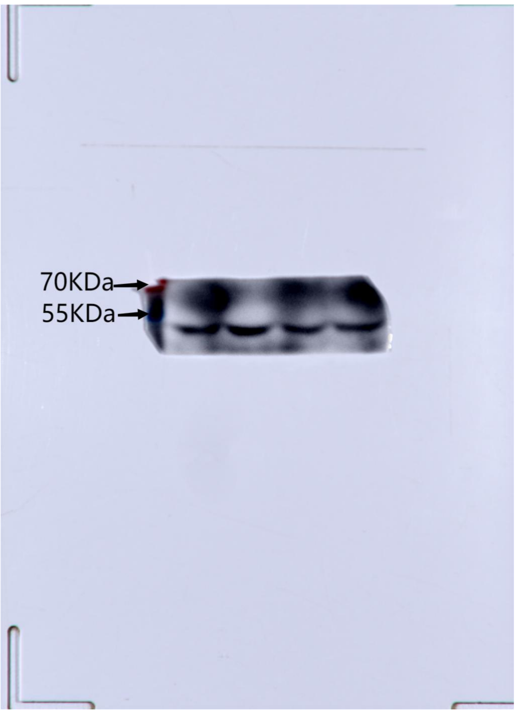

P-CaMKII

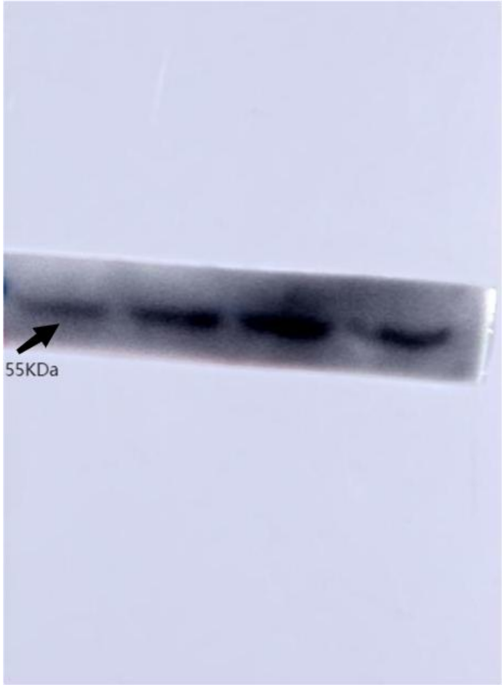

Figure 6 A

figure 6 A input $GABA_{B1}R$ et al(1)

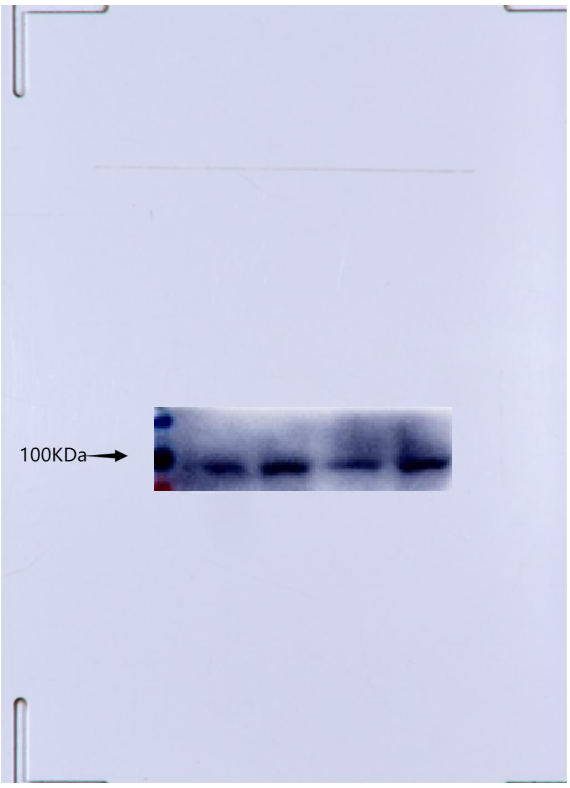

figure 6 A input pSer et al(1)

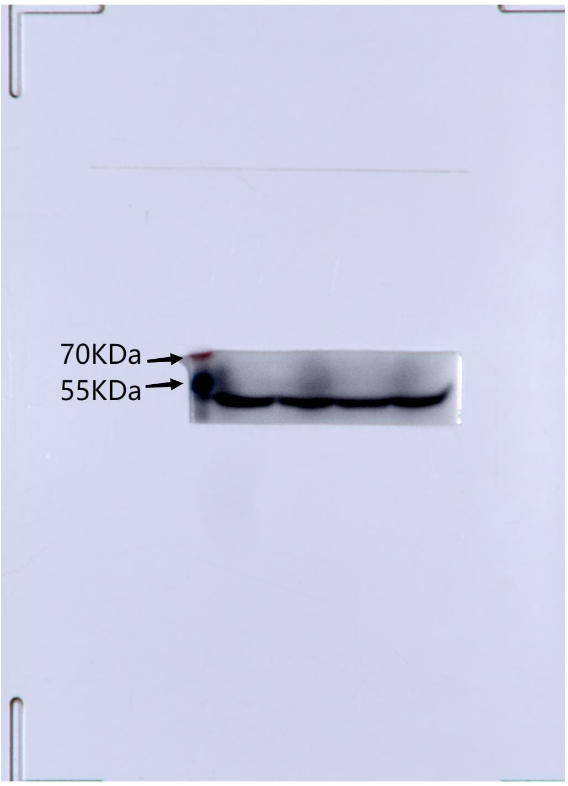

figure 6 A IP $GABA_{B1}R$ et al(1)

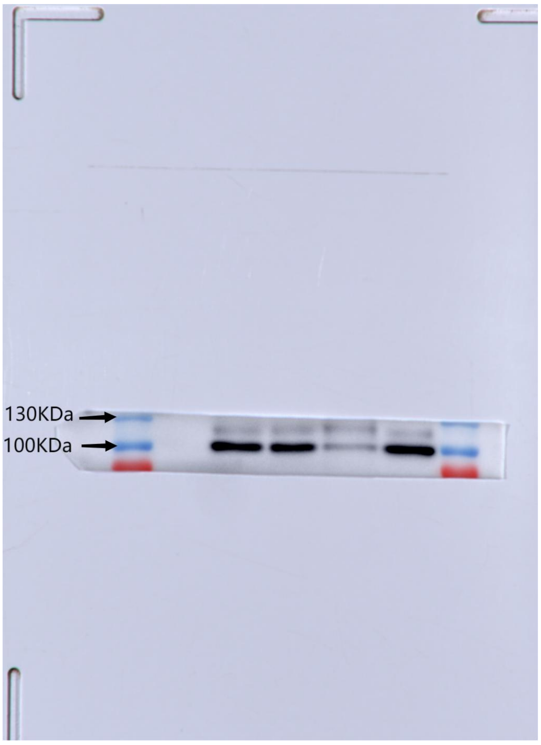

figure 6 A IP pSer et al(1)

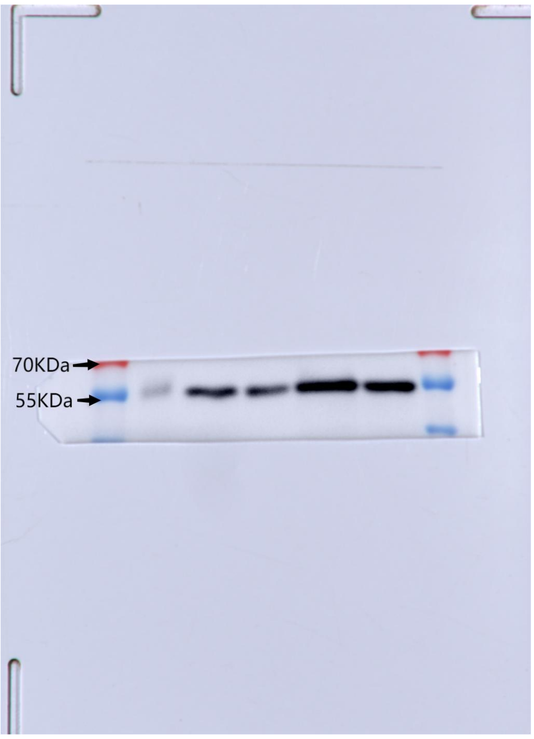

Figure 6 C

figure 6 c input GABA$_{B1}$R(1)

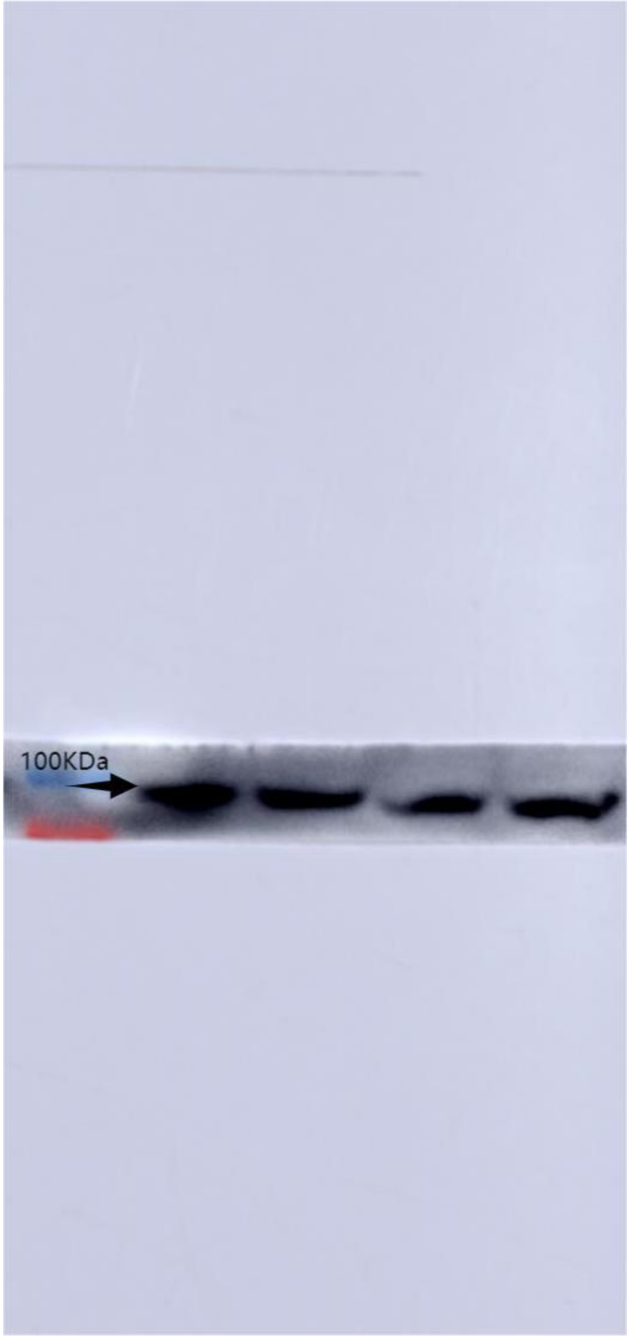

figure 6 C input p-CaMKII et al(1)

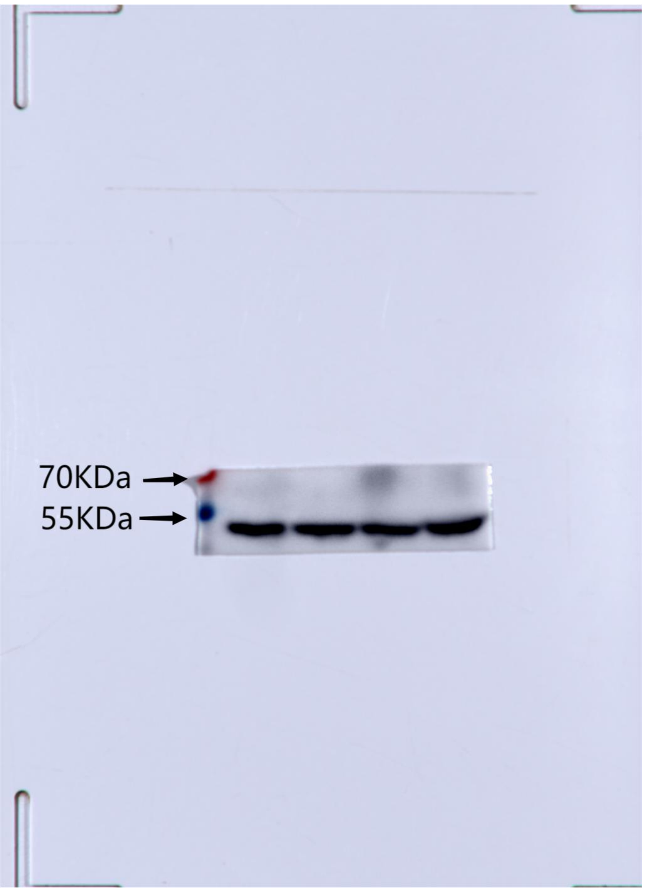

figure 6 c IP GABAB1R(1)

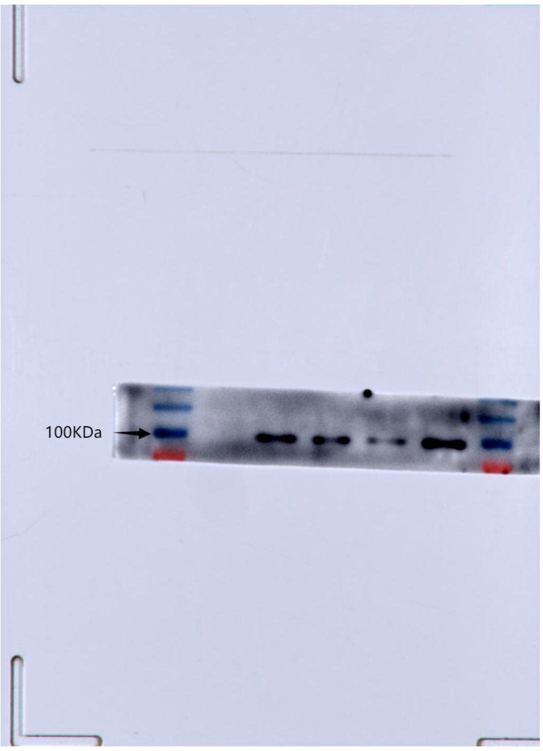

figure 6 C IP pCaMKII et al(1)

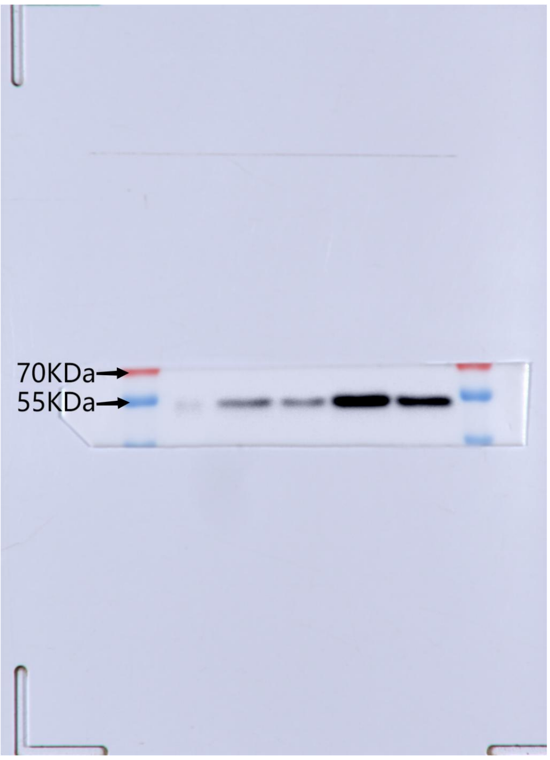

Figure 6 E

figure 6 E input CaMKII et al(1)

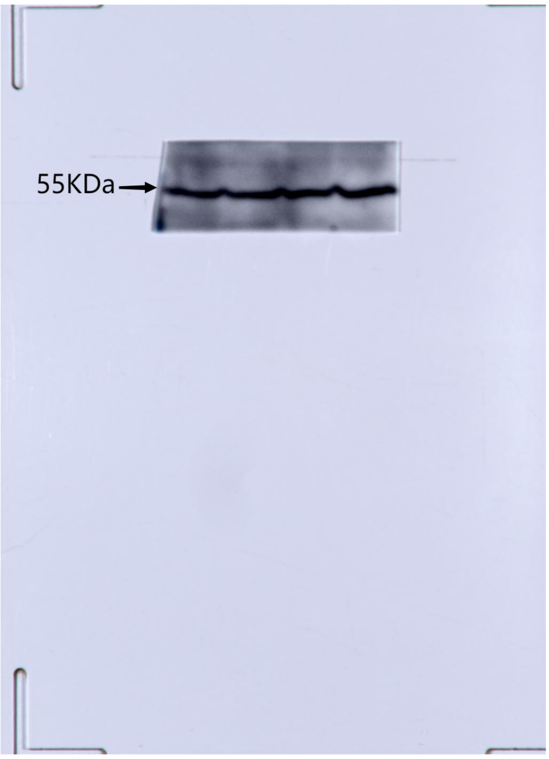

figure 6 E input GABAB1R et al(1)

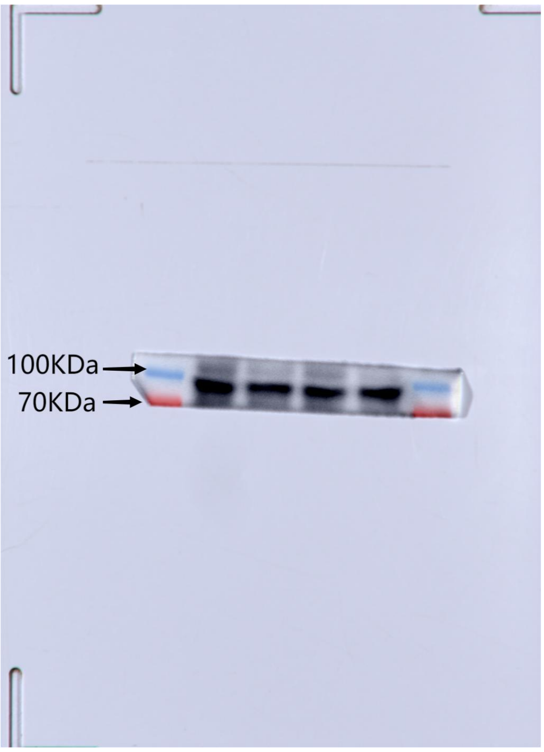

figure 6 E IP CaMKII et al(1)

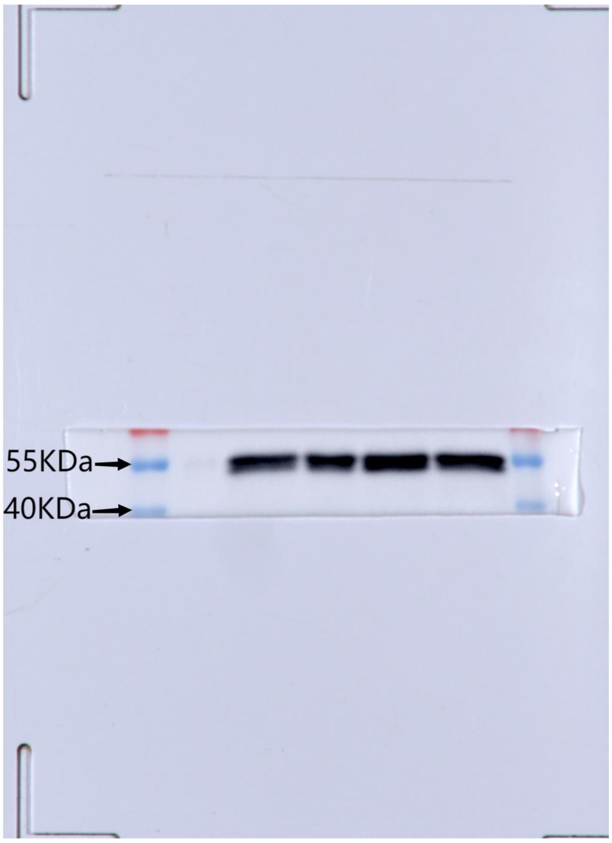

figure 6 E IP GABAB1R et al(1)

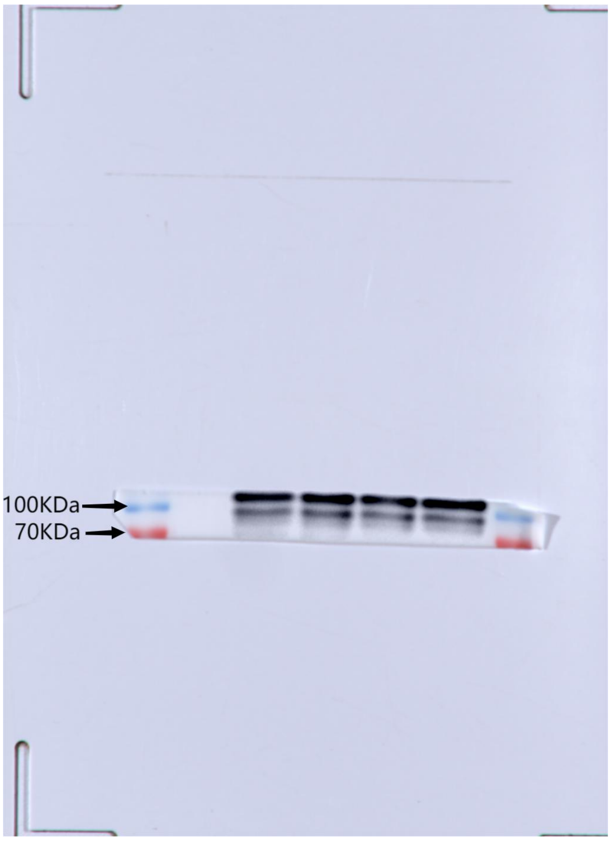

Supplement: Supplementary file 1 — Figures S1–S6 [file CNS-29-1345-s001.pdf]
